# The degree of cross-linking of polyacrylic acid affects the fibrogenicity in rat lungs

**DOI:** 10.1038/s41598-025-87174-6

**Published:** 2025-01-28

**Authors:** Taisuke Tomonaga, Hiroto Izumi, Chinatsu Nishida, Kazuma Sato, Yuiko Nakamura, Toshiki Morimoto, Yasuyuki Higashi, Ke-Yong Wang, Hidenori Higashi, Takuma Kojima, Kazuo Sakurai, Jun-ichi Takeshita, Akihiro Moriyama, Kei Yamasaki, Kazuhiro Yatera, Yasuo Morimoto

**Affiliations:** 1 Department of Occupational Pneumology, Institute of Industrial Ecological Sciences, University of Occupational and Environmental Health, 1-1 Iseigaoka, Yahata-nishi-ku, Kitakyushu, Fukuoka 807-8555 Japan; 2 Department of Environmental Health Engineering, Institute of Industrial Ecological Sciences, University of Occupational and Environmental Health, 1-1 Iseigaoka, Yahata-nishi-ku, Kitakyushu, Fukuoka 807-8555 Japan; 3 Department of Respiratory Medicine, University of Occupational and Environmental Health, 1-1 Iseigaoka, Yahata-nishi-ku, Kitakyushu, Fukuoka 807-8555 Japan; 4 Shared-Use Research Center, School of Medicine, University of Occupational and Environmental Health, 1-1 Iseigaoka, Yahata-nishi-ku, Kitakyushu, Fukuoka 807-8555 Japan; 5 Department of Chemistry and Biochemistry, The University of Kitakyushu, 1-1Wakamatsu-ku, HibikinoKitakyushu, Fukuoka 808-0135 Japan; 6 Research Institute of Science for Safety and Sustainability, National Institute of Advanced Industrial Science and Technology (AIST), 16-1 Onogawa, TsukubaTsukuba, Ibaraki 305-8569 Japan

**Keywords:** Polyacrylic acid, Cross-link, Intratracheal instillation, Pulmonary toxicity, Fibrosis, Rat, Respiratory tract diseases, Organic chemistry

## Abstract

Polyacrylic acid (PAA) with different concentrations of cross-linker was instilled into the trachea of rats to examine the effect of PAA crosslink density on lung disorders. Methods: F344 rats were intratracheally exposed to low and high doses of PAA with cross-linker concentrations of 0.1, 1.0, and 5.0% (CL0.1%, CL1.0%, and CL5.0%, respectively). Rats were sacrificed at 3 days, 1 week, 1 month, 3 months, and 6 months after exposure. PAA with different cross-linker concentrations caused an increase in neutrophil influx, cytokine-induced neutrophils, and chemotactic factor (CINC) in bronchoalveolar lavage fluid (BALF) from 3 days to 1 week after instillation. Lactate dehydrogenase (LDH) activity in BALF and heme oxygenase-1 (HO-1) release in lung tissue were higher in the CL0.1% exposure group during the acute phase. Lung histopathological findings also showed that severe fibrotic changes induced by CL0.1% were greater than those observed in CL1.0% and CL5.0% exposure during the observation period. CL0.1% was associated with more severe lung fibrosis, and a decrease in lung fibrosis was observed with increasing cross-linker concentrations, suggesting that the cross-link density of PAA is a physicochemical feature that affects lung disorders.

## Introduction

The types of respirable dust are broadly categorized into inorganic and organic substances. Pneumoconiosis, a well-known respiratory disease caused by respirable dust, is caused mainly by inorganic substances such as silica and asbestos, and is known to cause chronic inflammation, emphysematous changes fibrosis, and even lung cancer^1,2^. Organic substances, on the other hand, cause allergic diseases such as bronchial asthma and hypersensitivity pneumonitis, but they have not been thought to cause pulmonary fibrosis such as pneumoconiosis. In recent years, however, it has been reported that organic substances cause inflammation and fibrosis. In South Korea, there were many cases of progressive interstitial pneumonia among people who used disinfectants in humidifiers, which became a social problem^3,4^. In the United States, there were reports of e-cigarette users who developed acute respiratory failure^5,6^. In Japan, inflammation and fibrosis of interstitium around the airways have been reported in workers who handle water-absorbing cross-linked acrylic acid-based polymer compounds, which are used as intermediates for various products in daily life, such as diapers, cosmetics, shampoos, cosmetics, and food additives^7–10^. In the case of those workers, fibrosis progressed quickly, about two years after exposure to acrylic acid-based polymers, so the progression was faster than in lung damage caused by asbestos and crystalline silica, which are typical inorganic substances^7–9^. In animal exposure examinations, intratracheal instillation and inhalation exposure have been conducted with this acrylic acid-based polymer, and it has been reported that it causes lung disorders, including rapidly progressing fibrosis, similar to human cases^9,11,12^. In spite of these lines of evidence, however, the pathogenesis of lung disorders caused by acrylic acid-based polymers has not been elucidated.

PolyAcrylic acid (PAA) is composed of repeating chemical structural formulas of acrylic acid monomers, and the linear repeating structures of the monomers are intricately entangled. A cross-linker is used to cause the cross-linking of multiple linear molecules through covalent bonds. The cross-linking becomes more polymerized and forms a three-dimensional structure with complex meshes, and as the molecular weight and cross-linked structure increase, thickening and swelling by absorbing water are caused^13–15^. It is thought that the thickening and swelling properties associated with water absorption cause lung disorders.

We have previously conducted intratracheal installations on rats using PAAs with different molecular weights and cross-linked structures^16,17^. When evaluating the effects of the presence or absence of a cross-linked structure, it was observed that PAAs with a cross-linked structure caused higher lung injury and fibrogenicity than linear PAAs without a cross-linked structure^16^. This cross-linked structure is thought to be involved in biological effects since it is considered that water absorption and thickening properties change in a complicated manner by changing the density of the cross-linked structure. In this study, we aimed to investigate how changes in the degree of cross-linking structures of PAA affect pulmonary inflammation and fibrosis progression, using a rat model assuming human exposure level. We synthesized three types of PAAs with different cross-linking densities by varying the concentration of cross-linker and intratracheally instilling them into rats.

## Results

### Characterizations of polyacrylic acid with different degrees of cross-linking

**Fig. 1:**
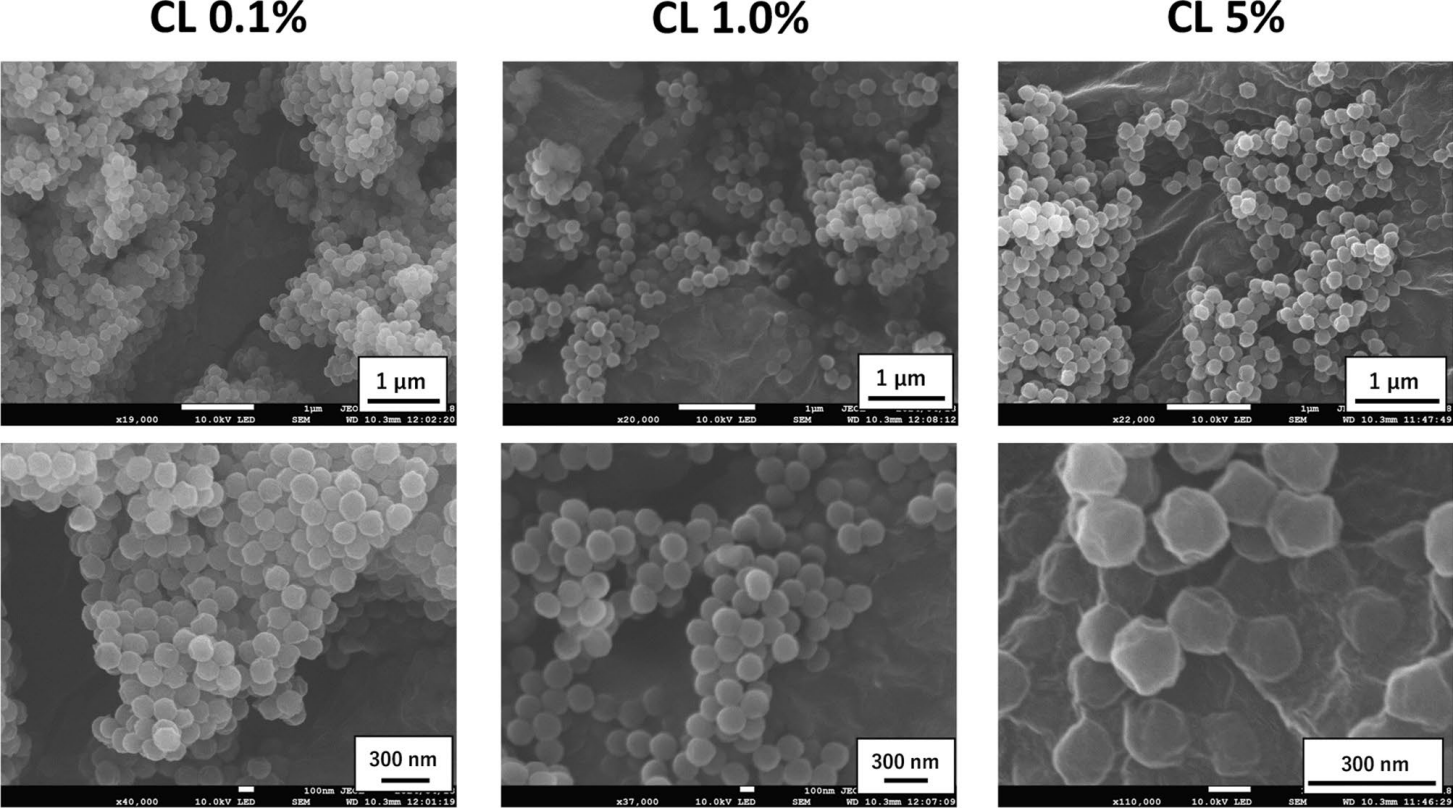
The images of scanning electron microscopy of the powder of polyacrylic acid with different concentrations of cross-linker. Uniform spherical shapes were observed for the PAA with CL0.1%, CL1.0%, and CL5.0%.

The fundamental characteristics of different cross-linking concentrations of PAA are summarized in Table 1. Figure 1 shows the scanning electron microscopy (SEM) by HITACHI S-4500 (Hitachi, Ltd., Tokyo, Japan) of the powder of the PAAs (Fig. 1). We examined the fundamental characteristics of the PAAs by preparing the molecular dispersion as follows: the polymers were dissolved in 0.1 M carbonate-bicarbonate buffer, then the solutions were alkalized with 2N NaOH and then neutralized with 1N HCl. The PAA without a cross-linking reagent in the same synthetic process had an average molecular weight (MW) of 50.4 × 10^5^ as measured by gel permeation chromatography (GPC) (a Prominence 501 system coupled with Dawn-Heleos-II(Wyatt Technology Europe GmbH, Dernbach, Rheinland-Pfalz, Germany)) using GF-7MHQ (Showa Denko K.K., Tokyo, Japan) with 0.1 M carbonate-bicarbonate buffer as the eluent^18,19^. The PAAs with cross-linking could not be measured, however, because the molecular weight was too large. On the other hand, the radius of gyration (Rg) in molecular dispersion measured by GPC was increased in a cross-linker concentration-dependent manner. Rg indicates the extent of a polymer, which is influenced by its molecular weight and polymer structure.

**Table 1:** Physiochemical characterization of the polymer used in the present study.

Physiochemical characterization	CL 0.1% Polyacrylic acid	CL 1.0% Polyacrylic acid	CL 5.0% Polyacrylic acid
Structural formula			
Cross-linker concentration (%M)	0.1	1.0	5.0
Radius of gyration (Rg) (nm)	30	217	276

### Body and lung weights

**Fig. 2:**
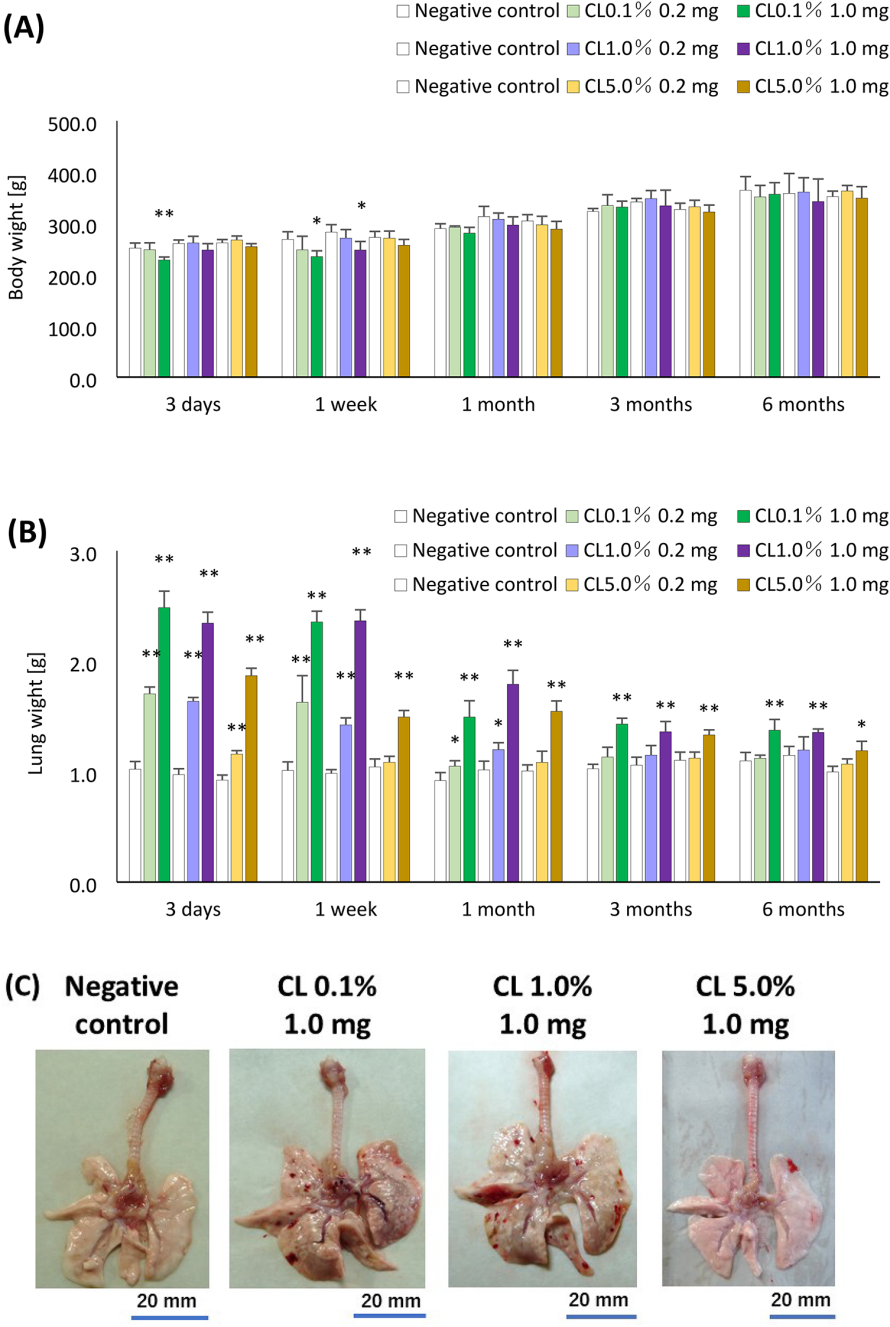
Observation of Body weight and lung findings after the instillation. (**A**) Body weight at each time after the instillation of PAAs with different cross-linker concentrations. (**B**) Lung weight at each time after the instillation of PAAs with different cross-linker concentrations. (**C**) Macro findings at 1 week after the instillation. There was a significant increase in body weight in CL0.1% and CL1.0% at 3 days or 1 week after exposure. In all PAA-exposure groups, a significant increase in lung weights was sustained throughout the observation period compared to the control group. The lungs in the CL0.1% and CL1.0% exposed groups showed swelling at 1 week after intratracheal instillation. Data are presented as mean ± SD for n = 5/group (vs each negative control *p < 0.05, **p < 0.01).

Body weight decreased dose-dependently at 3 days to 1 week after CL0.1% and CL1.0% exposure (Fig. 2A). Lung weights increased dose-dependently at each observation point in all of the PAA-exposure groups (Fig. 2B). During the acute phase, there was less lung swelling in the 1.0 mg group in a cross-linking-concentration dependent manner (Fig. 2C).

### Cell analysis and cell injury markers in bronchoalveolar lavage fluid (BALF)

**Fig. 3:**
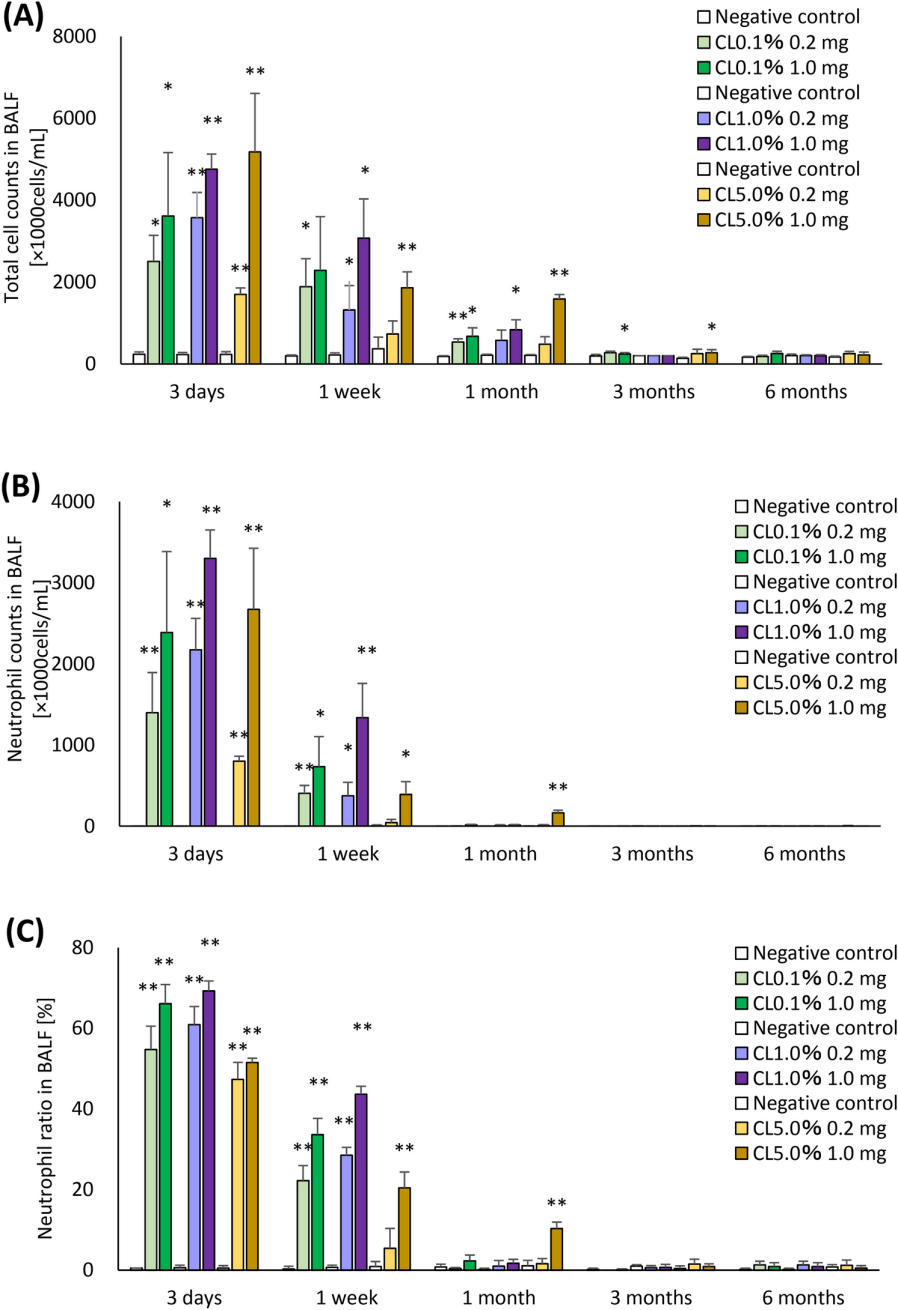

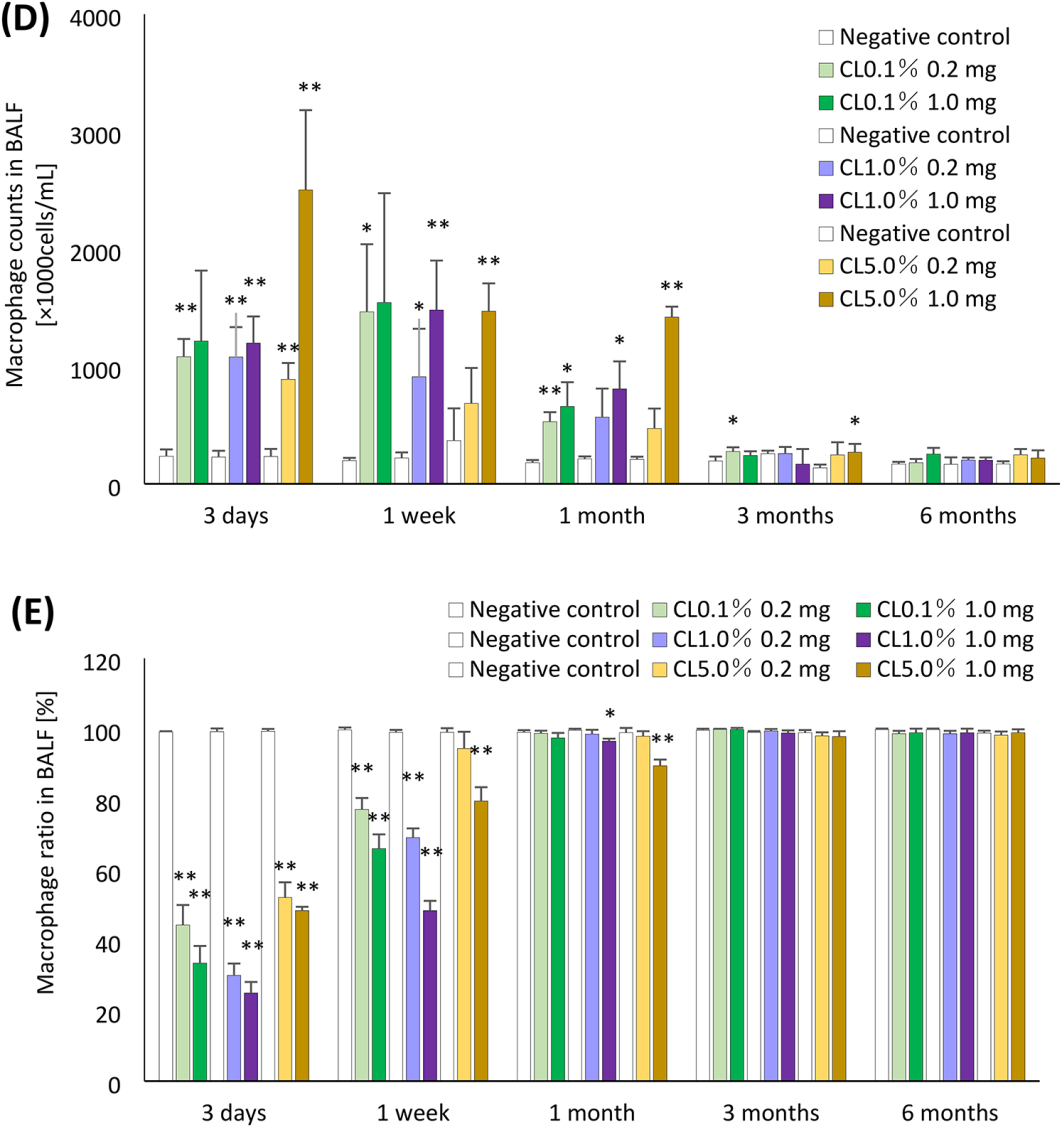
Analysis of cell number in BALF following intratracheal instillation of PAAs with different cross-linker concentrations. (**A**) total cell number in BALF. (**B**) neutrophil count in BALF. (**C**) percentage of neutrophils in BALF. (**D**) macrophage count in BALF. (**C**) percentage of macrophage in BALF. Total cell, neutrophil and macrophage counts in BALF in all of the exposed groups were higher than those in the control groups in a dose-dependent manner at 3 days to 1 month after exposure. Data are presented as mean ± SD for n = 5/group (vs each negative control *p < 0.05, **p < 0.01).

The results of inflammatory cell counts in BALF are shown in Fig. 3. There were significant increases in the number of total cells in all of the 1 mg-exposure groups from 3 days to 1 month after exposure compared to each control group, and a significant increase in the number of total cells was observed until 3 months after exposure in the CL0.1% and CL5.0% -high exposure groups (Fig. 3A). The number and percentage of neutrophils were significantly increased from 3 days to 1 week after exposure in CL0.1% and CL1.0% in both doses of 0.2 mg and 1.0 mg, and the exposure to 1.0 mg of CL5.0% induced a persistent increase until 1 month (Fig. 3B, C).

In the 1.0 mg exposure group of CL5%, the number and percentage of macrophages were higher 3 days after exposure compared to those of CL0.1% and CL1.0%, and these increase tendencies persisted until 3 months after exposure in the 1.0 mg exposure group of CL5% (Fig. 3D, E). In the acute phase, the increase in inflammatory cells in BALF was similar, regardless of the degree of cross-linking of PAA, but there was a difference in the persistence of increased inflammatory cells in BALF in CL0.1%, CL1.0%, and CL5.0%.

### Measurements of inflammatory markers in BALF and lung tissue

**Fig. 4:**
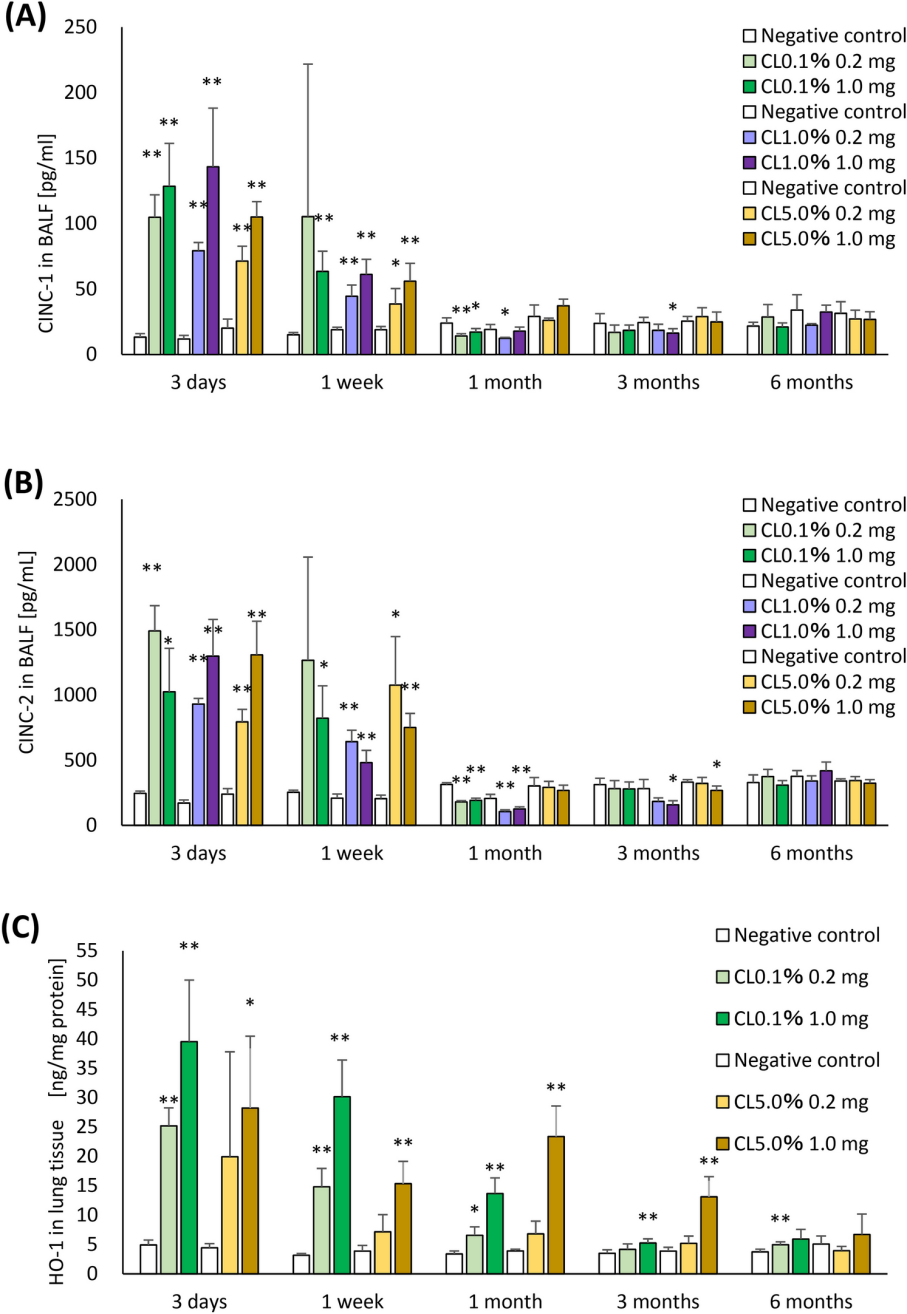
Analysis of cytokines in BALF and oxidative stress in lung tissue after intratracheal instillation. (**A**) CINC-1 concentration in BALF. (**B**) CINC-2 concentration in BALF. (**C**) HO-1 concentration in lung tissue. There was an increase of CINC-1 and CINC-2 in BALF in all exposure groups from 3 days to 1 week. HO-1 in lung tissue as an oxidative stress marker increased in a dose-dependent manner from 3 days to 1 month in all exposure groups, and had a tendency of increase in the 1.0 mg exposure group of both CL0.1% and CL5.0% until 3 months after exposure. Data are presented as mean ± SD for n = 5/group (vs each negative control *p < 0.05, **p < 0.01).

The concentrations of cytokine-induced neutrophil chemoattractant (CINC) -1 and CINC-2 in BALF, and the concentrations of heme oxygenase (HO)-1 in lung tissue following the intratracheal instillation of PAAs are shown in Fig. 4. CINC-1 and CINC-2 were increased in the exposure groups from 3 days to 1 week after exposure. HO-1 in lung tissue showed a tendency of significant increase in the 1.0 mg exposure group until 3 months after exposure in CL0.1% and CL5.0%, but at 1 month and 3 months after exposure HO-1 was more increased in CL5.0% than in CL0.1%.

### Cell injury markers in BALF

**Fig. 5:**
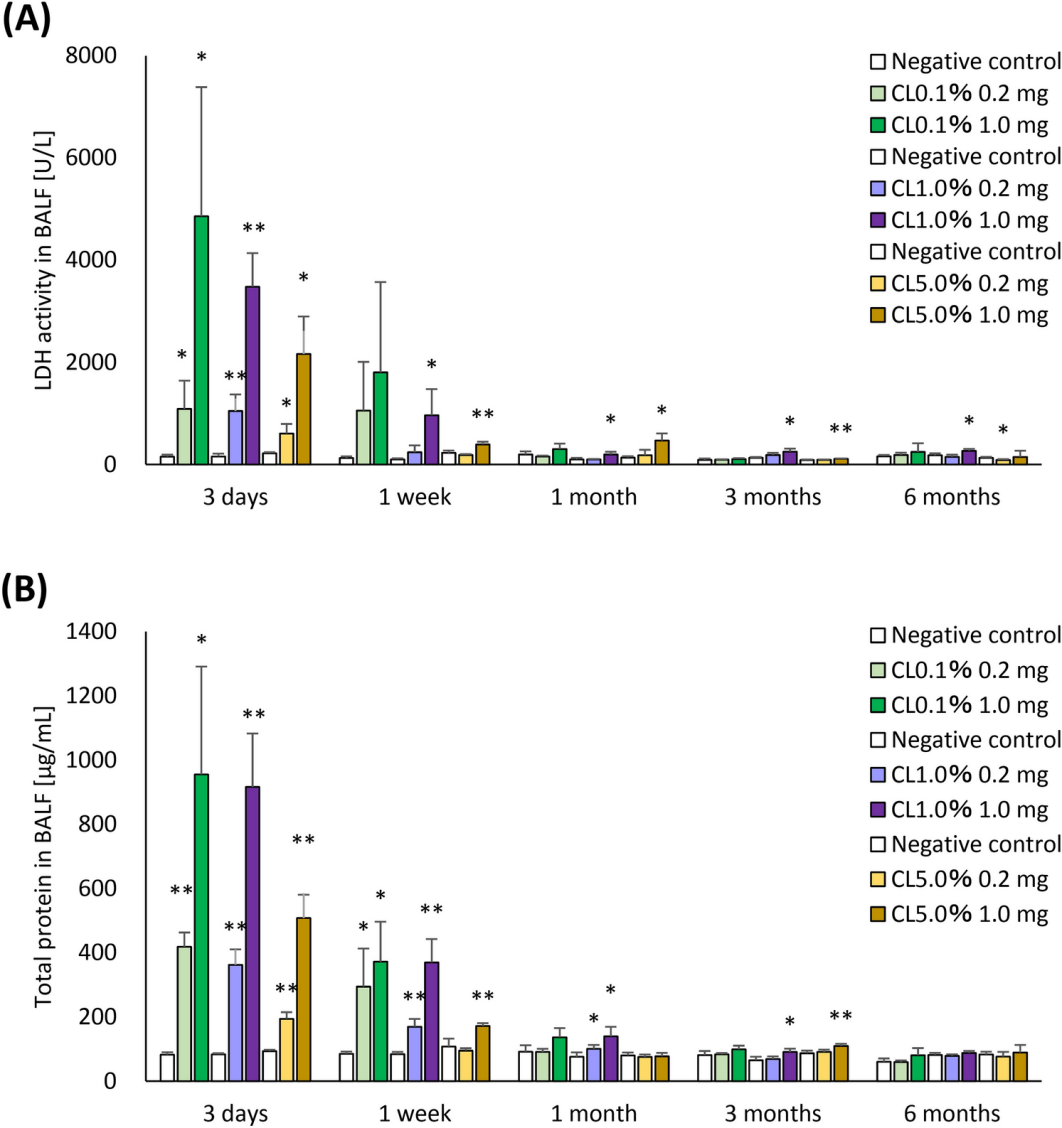
Analysis of released LDH activity and total protein in BALF following intratracheal instillation of PAAs with different cross-linker concentrations. (**A**) Released LDH activity in BALF. (**B**) Concentration of total protein in BALF. Released LDH activity in BALF in CL0.1% groups was higher than CL1.0% and CL5.0% groups at 3 days to 1 month after exposure, but there was a persistent increase of LDH activity and total protein concentration from 1 to 3 months. Data are presented as mean ± SD for n = 5/group (vs each negative control *p < 0.05, **p < 0.01).

The results of lactate dehydrogenase (LDH) activity and total protein in BALF are shown in Fig. 5. The results of LDH activity and the concentration of total protein in all of the exposure groups also showed tendencies of increase from 3 days to 1 month after exposure compared to each control group. The increase of the LDH activity was highest in CL0.1% and lowest in CL5.0%, showing cross-linking concentration-dependent changes (Fig. 5A). The increase in total protein concentration also tended to be lowest in CL5.0% (Fig. 5B).

### Histopathological features in the lung

**Fig. 6:**
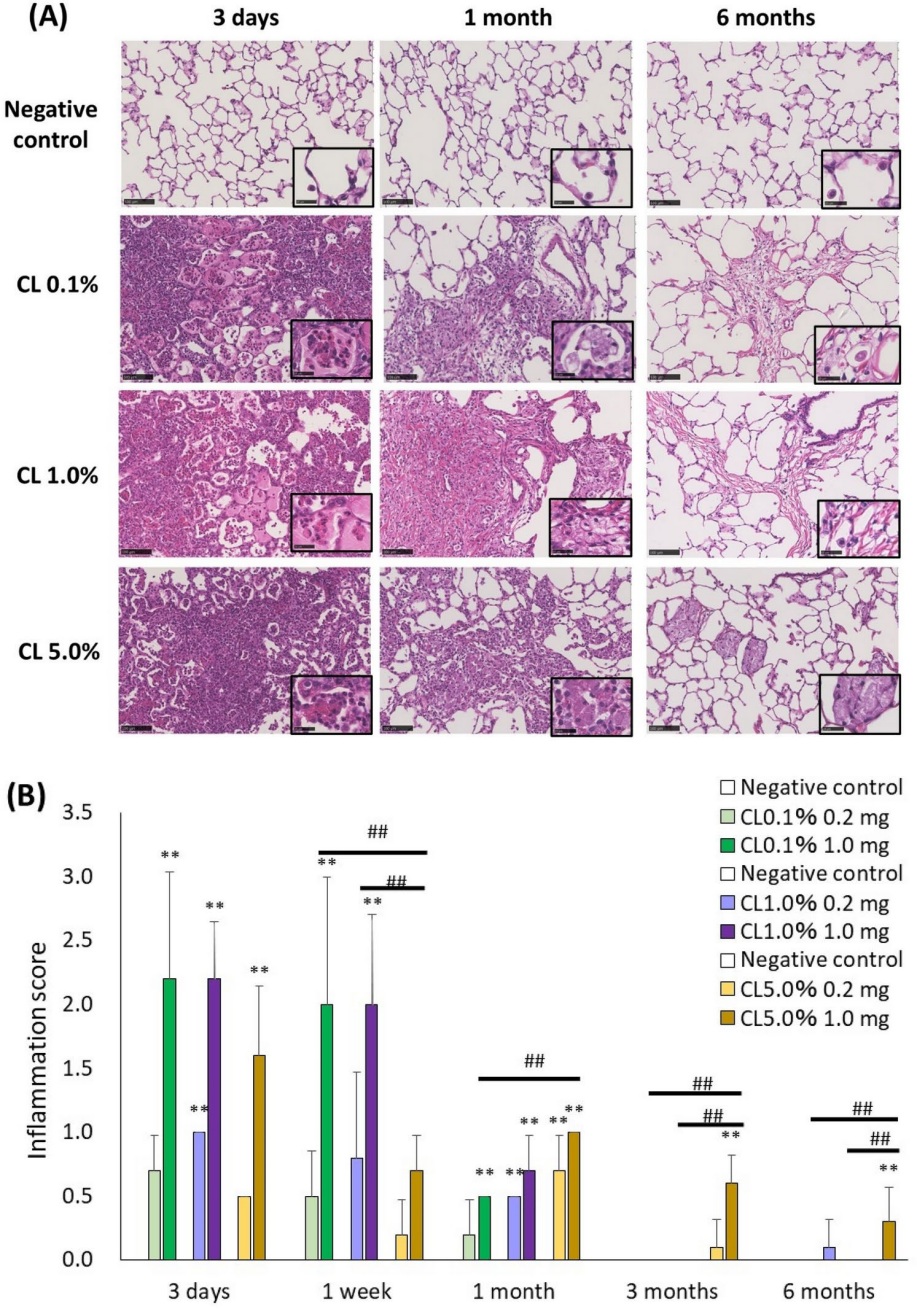
Histopathological findings of HE staining in lung-exposed PAAs with different cross-linker concentrations. (**A**) Histopathological findings in the lung at 3 days, 1 month, and 6 months after exposure of CL0.1%, CL1.0%, and CL5.0%. (**B**) Inflammation score in histopathological findings in the lung. There were severe inflammatory cell infiltrations into the alveoli, mainly neutrophils, which were remarkable in the lung in a dose-dependent manner 3 days after exposure to all of the PAAs. Inflammatory changes with fibrosis were observed from 3 days to 1 week after exposure (vs each negative control *p < 0.05, **p < 0.01, and vs each 1.0 mg exposure group #*p* < 0.05, ##*p* < 0.01).

**Fig. 7:**
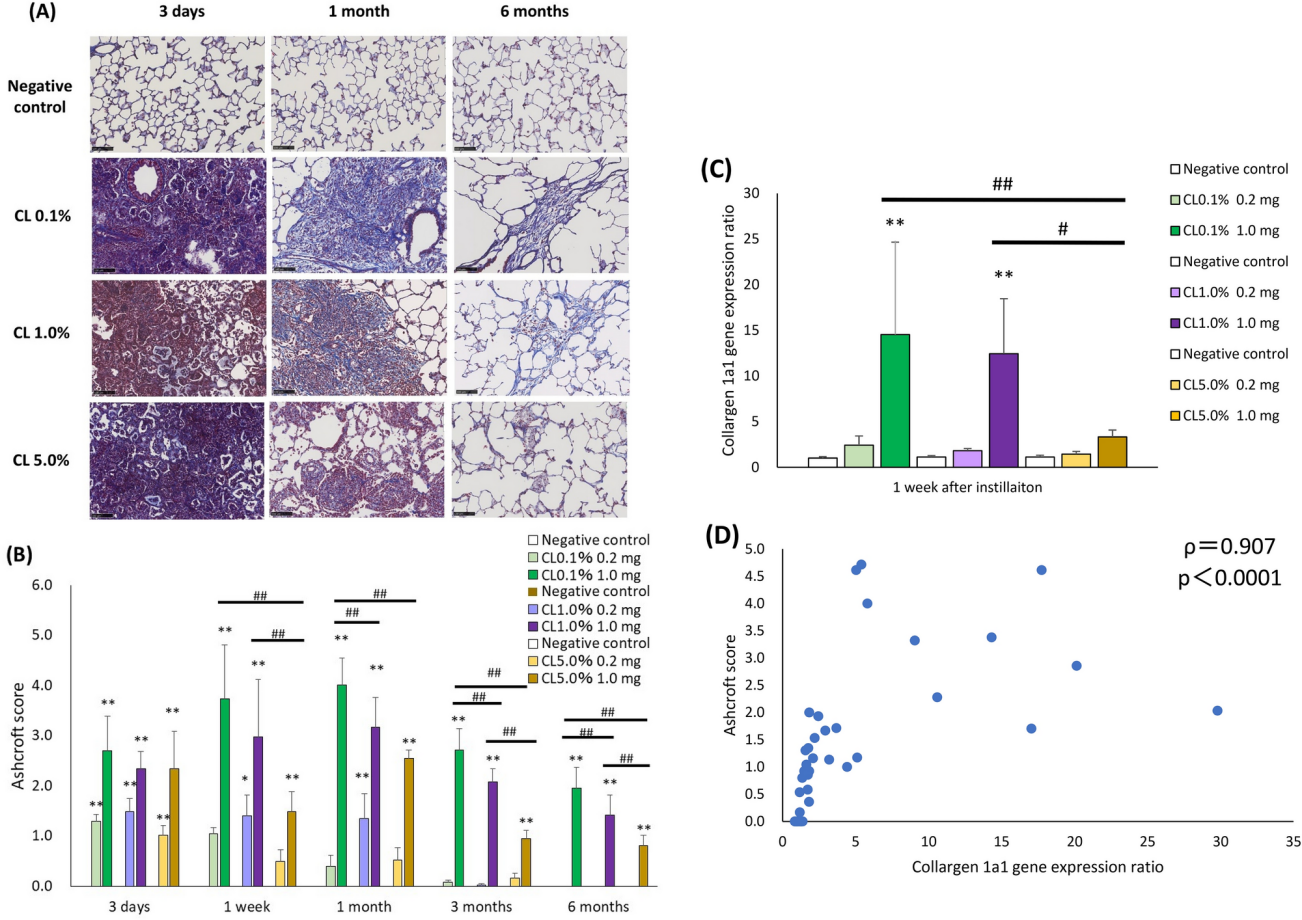
Histopathological findings of MT staining in lung-exposed PAAs with different cross-linker concentrations. (**A**) Histopathological findings in the lung at 3 days, 1 month, and 6 months after exposure of CL0.1%, CL1.0%, and CL5.0%. (**B**) Ashcroft score, which is the indicated fibrosis score, in histopathological findings in the lung. (**C**) Quantitative real-time polymerase chain reaction (qRT-PCR) in lung tissues at 1 week after intratracheal instillation on collagen 1a1 gene expression. (**D**) The relationship between lung fibrosis and collagen 1a1 gene expression at 1 week after exposure of PAAs. There was fibrosis in the 1.0 mg exposure group of all of the PAAs, whereas fibrosis was more spread in the 1.0 mg exposure group of CL0.1%. Evaluation by the Ashcroft score showed a dose-dependent increase in the score in the exposure of CL0.1%, CL1.0%, and CL5.0%. There were higher scores in the 1.0 mg exposure group of CL0.1% than in those of CL1.0% and CL5.0%. Evaluation by the collagen 1a1 gene expression showed a cross-linker concentration-dependent decrease in the score in the exposure of CL0.1%, CL1.0%, and CL5.0%. There was good correlation between Ashcroft score and collagen 1a1 gene expression. Data are presented as mean ± SD for n = 5/group (vs each negative control *p < 0.05, **p < 0.01, and vs each 1.0 mg exposure group #*p* < 0.05, ##*p* < 0.01).

Representative histopathological findings of the lungs at 3 days, 1 month, and 6 months after exposure to PAA with different cross-linking concentrations are shown in Fig. 6. There was inflammatory cell infiltration, mainly by neutrophils, into the alveoli, which was evident in a dose-dependent manner at 3 days after exposure. Inflammatory changes and an increase of fibroblast were observed from 3 days to 1 week after exposure, suggesting severe lung disorders. In particular, inflammatory cell aggregation was more pronounced in the 1.0 mg CL0.1% and CL1.0% exposure groups than in the CL5.0% group (Fig. 6A). In the evaluation by inflammation score, a dose-dependent increase in the score was observed in CL0.1%, CL1.0%, and CL5.0%. The CL0.1% 1.0 mg exposure group tended to have a higher score than the CL5.0% group (Fig. 6B). On the other hand, the inflammation score in the CL5.0% 1.0 mg exposure group was persistently increased compared to the CL0.1% and CL1.0% groups. Fibrosis in the CL0.1% 1.0 mg group, as compared to CL1.0% and CL5.0%, was the most severe and progressed the fastest, and the extent of fibrosis decreased in a cross-linker concentration-dependent manner (Fig. 7A). Ashcroft score assessment showed a dose-dependent increase in scores with PAA exposure. The CL0.1% 1.0 mg exposure group had a higher score than the CL1.0% and CL5.0% groups (Fig. 7B). To evaluate the validity of the Ashcroft score, we analyzed the quantitative real-time polymerase chain reaction (qRT-PCR) on the collagen 1a1 gene in lung tissues 1 week after intratracheal instillation. There was the same tendency of Ashcroft score that the CL0.1% in 1.0 mg exposure group had a higher expression than the CL1.0% and CL5.0% groups (Fig. 7C). Moreover, a good correlation between the Ashcroft score and collagen 1a1 gene expression was observed (Fig. 7D).

### The relationship between lung fibrosis and lung injury

**Fig. 8:**
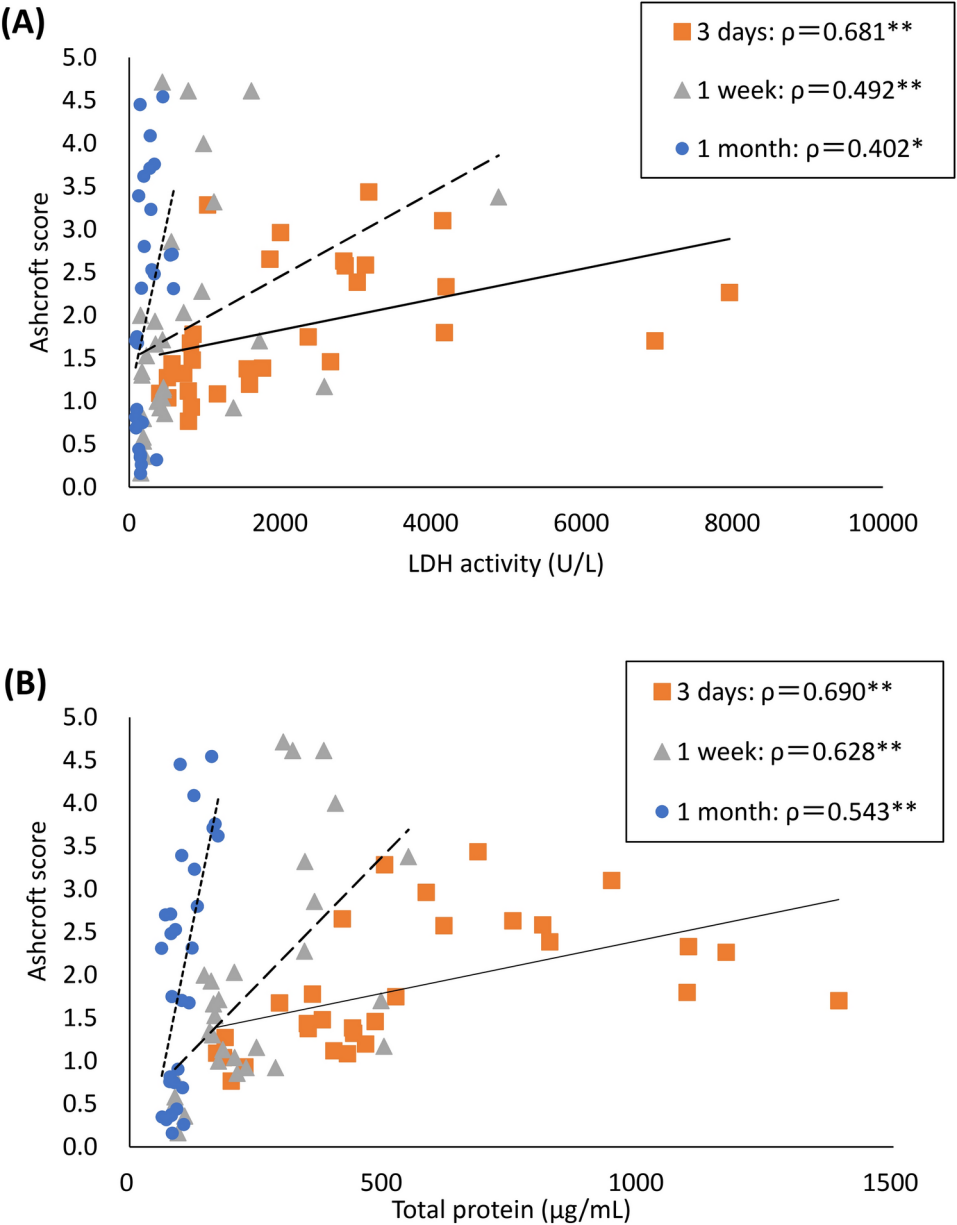
The relationship between lung fibrosis and lung injury from 3 days to 1 month after exposure to PAAs. (**A**) The relationship between lung fibrosis and release LDH activity. (**B**) The relationship between lung fibrosis and total protein concentration. There were good correlations between lung fibrosis and LDH activity or total protein as lung injury indicators from 3 days to 1 month after exposure. Values of ρ are Spearman’s rank correlation coefficient for the data at each time after exposure PAAs (significance level *p < 0.05, **p < 0.01).

The relationship between pulmonary fibrosis and lung injury is shown in Fig. 8. The data used is from 3 days to 1 month after exposure. LDH activity and total protein concentration as lung injury markers showed good correlations with the Ashcroft score, a fibrosis marker, suggesting that lung injury is related to the progression of fibrosis.

## Discussion

In this study, we used PAAs synthesized with different concentrations of cross-linkers, and intratracheal instillation was conducted on rats. Intratracheal instillation was used to evaluate the pulmonary toxicity of these PAAs. We estimated the human exposure equivalent to the maximum dose of 1 mg in this study by using the following formula that had been used previously^20^:

$$\begin{aligned} & \left( {{\text{amount}}\;{\text{of}}\;{\text{PAA}}} \right) = \left( {{\text{exposure}}\;{\text{concentration}}\;{\text{of}}\;{\text{particle}}} \right) \times \left( {{\text{tidal}}\;{\text{volume}}} \right) \times \left( {{\text{breathing}}\;{\text{frequency}}} \right) \\ & \quad \times \left( {{\text{exposure}}\;{\text{hours}}\;{\text{in}}\;{\text{a}}\;{\text{day}}} \right) \times \left( {{\text{particle}}\;{\text{deposition}}\;{\text{fraction}}} \right) \\ \end{aligned}$$

Assuming that the lung deposition rate (0.1) of PAA is the same for humans and rats if humans were exposed to the TLV-TWA (Threshold Limit Value—Time-Weighted Average from American Conference of Governmental Industrial Hygienist; ACGIH) level of general dust (3 mg/m^3^), this would correspond to an exposure period of approximately 926 days (calculation in rat and human under assumption of tidal volume 2.1 and 625 mL/times; breathing frequency volume 102 and 12 times/min; and exposure hours in day 6 h (lung weights of rats and humans are set at 1 g and 1000 g, respectively)). Assuming that a worker continued to work under conditions of 3 mg/m^3^ (8 h a day, 5 days a week), a high dose of 1 mg instilled into the trachea would be equivalent to an exposure period of approximately 3.6 years. In actual worker cases, lung disorder has been reported after exposure to acrylic acid-based polymers for more than two years^8^; we considered that the level of exposure in the present study corresponded to the real environment. Although intratracheal instillation is useful for evaluating the potential for inflammation and fibrosis, it is important to note that it is a non-physiological exposure method and involves a single exposure, and therefore there is a possibility that intratracheal instillation does not completely reproduce the pathological conditions expected in humans.

In this study, weight loss, an indication of the overall condition, was observed in the acute phase in CL0.1% and CL1.0%, and the inflammation score and HO-1 concentration in lung tissue in lung pathological findings tended to be higher in CL0.1%. However, in BALF analysis, the acute increase in neutrophil count, neutrophil percentage, and CINC-1 and CINC-2, which are chemotactic factors for neutrophils released from alveolar macrophages, was observed to be similar in all PAA crosslinking levels. Surprisingly, after the acute inflammatory changes, fibrosis progressed to a severe stage in CL0.1% and CL1.0%, while fibrosis remained mild in CL5.0%, indicating that the potential of fibrosis formation differed depending on the crosslinking concentration. Pathological findings of the lungs revealed infiltration of inflammatory cells into the alveolar space and proliferation of fibroblasts in CL0.1% and CL1.0% mice. This suggests that exposure to CL0.1% and CL1.0% induced severe pulmonary inflammation followed by rapidly progressing fibrotic changes. We think the results of lung swelling in the CL0.1% and CL1.0% exposure groups and increased collagen gene expression in lung at 1 week after exposure reflect the pathological findings. Considering that inflammation leads to subsequent fibrosis in lung disorders caused by inorganic substances^21,22^, it is thought that PAAs have a strong fibrogenicity after inflammation. The development of fibrosis after acute inflammation has been observed in animal exposure studies and clinical manifestations, and is common in LPS exposure models that induce acute inflammation and in ARDS with acute respiratory failure^23–25^. Acute respiratory failure in animal models due to LPS or bleomycin causes severe inflammation of the lungs that ends in 1–2 weeks and causes fibrosis without long-term inflammation^24,25^.

In the worker cases, fibrosis was observed about two years after the start of exposure to an acrylic acid-based polymer^10^ and it progressed faster than with asbestos or silica. Considering that acrylic acid-based polymers used in products often demand water-soluble applications and the cross-linkers are generally used at a concentration of about 0.1%, the early observation of fibrosis in the CL0.1% in this study is not inconsistent with the pathology in humans. On the other hand, fibrosis progressed only mildly with the CL5.0%, but inflammation tended to persist. Considering that persistent inflammation leads to subsequent fibrosis in lung disorders caused by inorganic substances^20,26^, there is a possibility that, in the long term, fibrosis may progress or lead to irreversible lesions such as tumors. The inflammatory and fibrotic potential of the CL5.0% may be lower than that of CL0.1% and CL1.0%, but it is considered to be high among general inhalable chemicals.

The relationship between the crosslink density of the PAAs and fibrosis is considered to be as follows. It is known that the water absorption of PAA increases when the polymer has a cross-linked structure, but the water absorption decreases when the crosslink density increases beyond a certain level^14,27,28^. The water absorbency of PAA may cause biological hyperosmotic stress on an organism. Hyperosmotic stress on an organism is reportedly associated with various pathological conditions such as induction of inflammatory cytokines and apoptosis^29–32^. Schwartz et al. reported that when three epithelial cell lines (colorectal HT29, bladder T24, and lung A549) were exposed to glycol-derived compounds, the presence of hyperosmolar concentrations induced significant production of the proinflammatory cytokines IL-6, IL-8, TNF-α, and IL-1β in all cell lines^31^. Singh and Ramarao also reported that the culture medium of RAW cells with mannitol induced cell death in an osmotic pressure-dependent manner^32^. Alveolar macrophages may be involved in the mechanism of fibrosis caused by PAA. We previously reported that alveolar macrophages had higher levels of BiP and CHOP, indicators of endoplasmic reticulum stress, than lung tissue after intratracheal instillation of PAA^33^. In addition, considering that an increase in TGF-β released from alveolar macrophages, which is involved in fibrosis formation, was observed after exposure to PAA^34^, it is speculated that when alveolar macrophages phagocytosed PAA, hyperosmotic stress damages the alveolar macrophages, inducing TGF-β, which is involved in fibrosis formation, and progressing to fibrosis. It is also possible that the polymer expands due to the inclusion of water, preventing clearance from the lungs, causing retention of the polymer in the lungs, and leading to atelectasis, which may have contributed to the progression of fibrosis. It is thought that airway obstructions observed in cross-linked water-soluble acrylic acid polymer exposure human report support the above consideration^10^. In our study, fibrosis was most severe at 0.1% CL, which is considered to be the highest water absorbency and stress, and fibrosis potential was reduced as the cross-linker concentration increased. Considering that a previous comparison of the pulmonary fibrotic potential of non-cross-linked and cross-linked (0.1% or less cross-linker) PAAs showed stronger fibrosis in the cross-linked PAA^16^, it is thought that the increased water absorption caused by the change in crosslink density causes fibrosis.

**Fig. 9:**
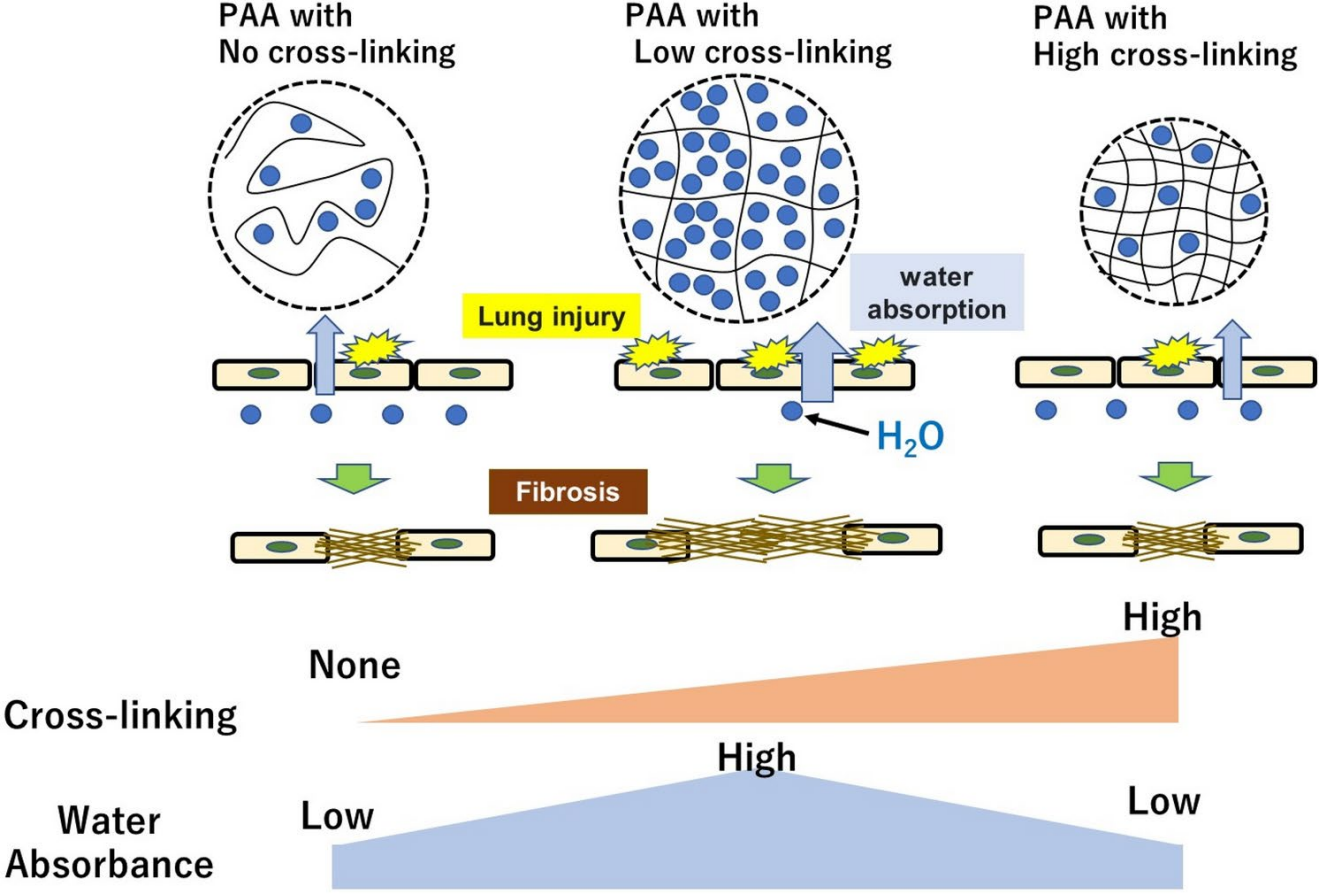
Schematic overview of the results of this study. PAA causes changes in osmotic pressure due to water absorption, and differences in water absorption by PAA with different degrees of cross-linking may have affected the differences in fibrosis via cell injury.

Furthermore, in the acute phase of this study, the LDH activity and total protein concentration in BALF, which are indicators of lung injury, were most elevated in the CL0.1% and tended to be the lowest in the CL5.0%. There are reports that severe lung injury leads to the progression of fibrosis. It has been reported in inhalation exposure and intratracheal instillation of MWCNT in mice that the increase in LDH in BALF and the amount of MWCNT deposition and fibrosis level showed a similar tendency of increase^35,36^. In an intratracheal instillation of bleomycin in mice, the increase in LDH activity in BALF and the progression of fibrosis were observed in a dose-dependent manner^24^. In the present study, a correlation was observed between lung injury and fibrosis from 3 days to 1 month after instillation (Fig. 8), so it is possible that the CL0.1%, which caused the highest lung injury, may have progressed to fibrosis early in the tissue repair process. As mentioned above, considering that the increased water absorption caused by changes in crosslink density leads to fibrosis, it is speculated that lung injury due to increased water absorption contributed to the progression of fibrosis (Fig. 9).

In this study, we analyzed the effects of PAAs with different cross-linking densities on the lungs by varying the cross-linker concentration. CL0.1% tended to cause the most severe inflammation and the strongest fibrosis in the acute phase, and a decrease in pulmonary fibrogenicity was observed as the cross-linker concentration was increased. It is thought that the water absorbency of PAA influences lung disorders since fibrogenicity decreases with increasing cross-linking concentration. Taken together, it is suggested that the crosslink density of PAA is a physicochemical feature that influences lung disorders.

## Methods

### Sample polymer

Using a previous method^37^, we synthesized a sample of PAA by polymerization of the acrylic acid monomer adding different cross-linker concentrations (0.1 mol%, 1.0 mol%, and 5.0 mol%) and labeled them CL0.1%, CL1.0%, and CL5.0%, respectively. The details of the preparations are described in the supplementary file (Figure S1). PAA, a white, easily scattered powder, was mixed with distilled water and slowly stirred for 40 min (Mag-Mixer MF820 or MD300, Yamato Scientific Co., Ltd., Tokyo, Japan).

### Animals

Male Fischer 344 rats (8 weeks old) (The Jackson Laboratory Japan, Inc., Kanagawa, Japan) were acclimated for 4 weeks at the Laboratory Animal Research Center of the University of Occupational and Environmental Health, Japan with commercial food and water available ad libitum. Finally, we used 12-week-old male Fischer 344 rats and performed intratracheal instillation. All procedures and animal handling were performed under the guidelines described in the "Japanese Guide for the Care and Use of Laboratory Animals" and approved by the Animal Experiment Committee of the University of Occupational and Environmental Health, Japan (Animal Research Ethics Approval Proposal No.; AE17-009). All methods were performed in accordance with the ARRIVE guidelines (https://arriveguidelines.org).

### Intratracheal instillation

Doses of 0.2 mg (0.8 mg/kg BW) and 1.0 mg (4.0 mg/kg BW) of PAA suspended in 0.4 mL of distilled water at different cross-linker concentrations were administered by single intratracheal instillation into the lungs of rats (12 weeks old). These maximum doses were set to avoid overloading the lungs in anticipation of human exposure, based on our previous studies^11,16,17^. A control group was established for each intratracheal instillation, with distilled water administered to the control group. Rats were instilled intratracheally under sevoflurane (Viatris Inc., Canonsburg, PA, USA) inhalation anesthesia. Briefly, the laryngeal extension was performed using a laryngoscope blade (MAC1, Rudolf Riester GmbH, Jungingen, Germany), and an animal feeding needle (KN-348, Natsume Seisakusho Co., Ltd., Tokyo, Japan) was inserted directly into the trachea and the suspension was instilled manually. Next, 3 mL of air was inserted into the trachea twice with a syringe from the animal feeding needle, to prevent choking due to the sample during intratracheal instillation. Although the procedure of adding air pressure is thought to have the potential for lung damage, there is an air leak around the cannula into the trachea, and the air was added with the airway open. No alveolar wall destruction has been observed in previous experiments^16,17^, so the possibility of barotrauma occurring is considered low. The rats were then spontaneously awakened and observed periodically.

### Animals following intratracheal instillation

Five rats were in each exposure and control group at each time point. Rats were dissected at 3 days, 1 week, 1 month, 3 months, and 6 months after intratracheal instillation. In brief, body weight, lung weight and amount of BALF were measured, and samples of BALF and lung tissue were collected at autopsy. Autopsies were performed as follows. Under isoflurane (Viatris Inc., Canonsburg, PA, USA) inhalation anesthesia, body weight was measured, blood was removed from the heart, and the blood in the lungs was perfused with saline solution. Lungs were extracted from the body, lung weights were measured. With the left main bronchus clamped, the right lung was inflated with saline at a pressure of 20 cm H_2_O, and the fluid (BALF) was collected in collection tubes by free fall. This process was performed twice, and BALF was collected in a total of seven to 14 mL. The amount of BALF recovered was affected by the growth of the rat lung and inflammatory changes in the exposed groups. However, the recovery was performed at constant pressure, and we confirmed that recovery within each group was stable. Then the right and left lungs were separated. The right lung after BALF collection was used for the measurement of HO-1, and the left lung was used for histopathological evaluation. The left lung was inflated and fixed with 10% formaldehyde under pressure of 25 cm H_2_O for histopathological evaluation.

### Cytospin analysis of inflammatory cells and measurement of inflammation-related markers in BALF

The BALF was used for analyses of inflammatory cells and inflammation-related markers in BALF. The BALF was centrifuged at 400 g for 15 min at 4 °C, and the supernatant was transferred to a new tube for determination of total protein, lactate dehydrogenase (LDH) and cytokines. Pellets, which were used for analysis of inflammatory cells, were washed in polymorphonuclear leukocyte (PMN) Buffer (137.9 mM NaCl, 2.7 mM KCl, 8.2 mM Na_2_HPO_4_, 1.5 mM KH_2_PO_4_ and 5.6 mM C_6_H_12_O_6_) in suspension and centrifuged at 400 g at 4 °C for 15 min. After the removal of the supernatant, the pellet was resuspended in 1 mL of PMN buffer. Cell counts in the resuspended samples were measured with ADAM-MC (AR BROWN CO., LTD., Tokyo, Japan), cells in the resuspended samples were spread on glass slides with cytospin, fixed, stained with Diff-Quik (Sysmex CO., Kobe, Hyogo, Japan), and the number of neutrophils and alveolar macrophages was measured by microscopic observation. LDH activities released into the BALF supernatant were measured with the Cytotoxicity Detection KitPLUS (LDH) (Roche Diagnostics GmbH, Mannheim, North Rhine-Westphalia, Germany) according to the manufacturer’s instructions. LDH activity was estimated using a standard curve obtained from known concentrations of recombinant LDH derived from rabbit muscle (Oriental Yeast Co., ltd., Tokyo, Japan). Protein concentrations in BALF supernatants were determined using the Pierce™ 660 nm Protein Assay (Thermo Scientific Inc., Rockford, IL, USA). Ford, Illinois, USA).

### Measurement of chemokine in BALF and HO-1 in lung tissue

Concentrations of CINC-1 and CINC-2 in BALF were measured with the ELISA kits #RCN100 and #RCN200 (R&D Systems, Minneapolis, MN, USA), respectively. All measurements were performed according to the manufacturer’s instructions. The third lobe of the right lung was cultured in T-PER tissue containing protein inhibitor cocktail (P8340, Sigma-Aldrich, St. Louis, MO, USA) and cOmplete Mini (Roche Diagnostics GmbH, Mannheim, Nordrhein-Westfalen, Germany), and homogenized in Protein Extraction Reagent (Thermo Scientific Inc., Rockford, IL, USA) and centrifuged (20,400 g at 4 °C for 10 min). Protein concentrations in the supernatant were determined with Pierce™ 660 nm Protein Assay Reagent (Thermo Scientific Inc., Rockford, IL, USA), using bovine serum albumin as a standard. HO-1 measurements with the Elisa kit ADI-EKS-810A (Enzo Life Sciences, Farmingdale, NY, USA) were corrected by the protein concentration in the supernatant to calculate the final HO-1 concentration in the lung tissue.

### Histopathology

Formaldehyde-fixed lung tissue was embedded in paraffin, sectioned at a thickness of 4 μm, and then stained with hematoxylin and eosin (HE) and Masson trichrome (MT) staining. The lung inflammation and fibrosis were examined using the inflammatory cell infiltration score^16,38^ and the Ashcroft score^39^, respectively, according to previous reports^11,16,38^. Briefly, the inflammatory cell infiltration score was calculated by scoring the degree of inflammatory cell infiltration in the lung tissue as none (0), minimal (0.5), mild (1), moderate (2), or severe (3). The mean and standard deviation of the scores were calculated for each group. Pulmonary fibrosis was assessed by scoring histopathological findings in the lungs on a scale of 0 to 8 using the modified Ashcroft score, with the mean and standard deviation calculated for each group.

### Total RNA extraction

The third lobes of the right lungs (n = 5 per group per time point) were homogenized while using a QIAzol lysis reagent with a TissueRupotor (Qiagen, Hilden, Ger-many). Total RNA from the homogenates was extracted using a miRNeasy Mini Kit (Qiagen, Hilden, Germany) following the manufacturer’s instructions. RNA purity and integrity were evaluated by ND-1000 Spectrophotometer (NanoDrop, Wilmington, USA), Agilent 2100 Bioanalyzer (Agilent Technologies, Palo Alto, USA).

### Validation of gene expression data using quantitative real-time polymerase chain reaction

Quantitative real-time polymerase chain reaction (qRT-PCR) was performed as described previously^38^. Briefly, the total RNA extracted from the lungs at each observation point in each group was transcribed into cDNA (High-Capacity cDNA Reverse Transcription Kit, Thermo Fisher Scientific Inc., MA, USA). qRT-PCR assays were performed while using TaqMan (TaqMan Gene Ex-pression Assays, Thermo Fisher Scientific Inc., Waltham, MA, USA) according to the manufacturer’s protocol. Gene expression data were analyzed by the comparative cycle time (ΔΔCT) method. The Assays-on-Demand TaqMan probes and primer pair were Col1a1 (Assay ID Rn01463869_g1). All experiments were performed in aStepOnePlusTM Real-Time PCR Systems (Thermo Fisher Scientific Inc., MA, USA). All expression data were normalized to endogenous control β-actin expression (Assay ID Rn00667869_m1) and calculated relative to their gene expression in each negative control.

#### Statistical analysis

Statistical analysis was performed using IBM SPSS Statistics version 25 (https://www.ibm.com/jp-ja/products/spss-statistics) (IBM Corporation, Chicago, IL, USA). *p*-values < 0.05 were determined to be statistically significant. The Dunnett and Tukey’s honestly significant difference (HSD) tests were appropriately used to detect rat differences between rats exposed to different cross-linker concentrations of PAA samples and the control group. Construct validity was measured using Spearman’s rank correlation coefficients (ρ) between the Ashcroft scores as lung fibrosis indicators and lung injury markers.

## Supplementary Information

Supplementary Information.

## Data Availability

The datasets during and/or analyzed during the current study are available from the corresponding author on reasonable request.
