# Supplementary material for: The degree of cross-linking of polyacrylic acid affects the fibrogenicity in rat lungs
Source: Sci Rep. 2025 Jan 28;15:3514. doi: 10.1038/s41598-025-87174-6 (PMC11775097; doi:10.1038/s41598-025-87174-6)
Supplement: Supplementary file 1 — Supplementary Information. [file 41598_2025_87174_MOESM1_ESM.docx]

Supplementary material

Figure S1


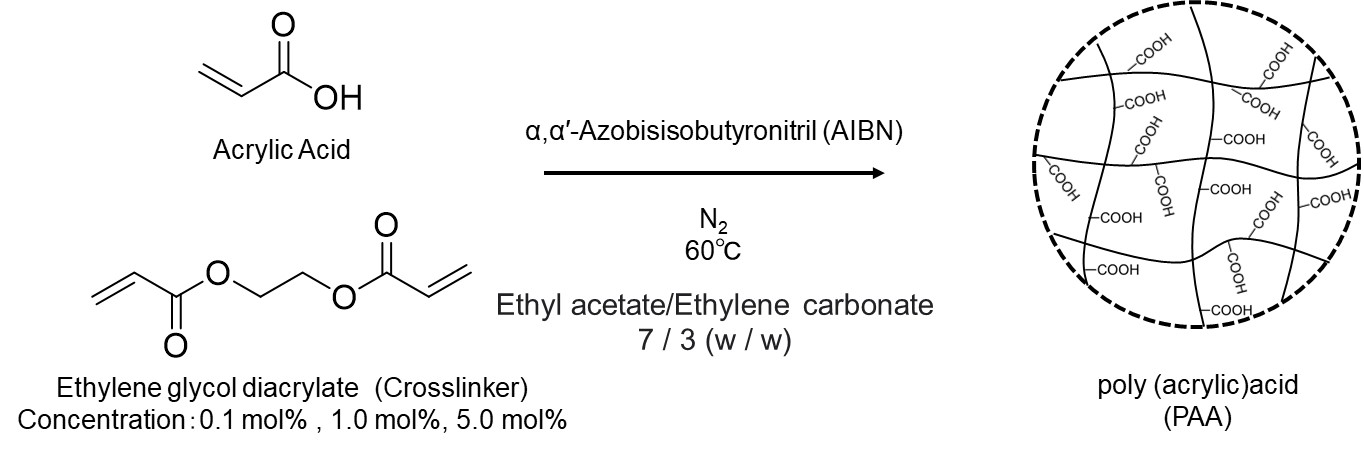


Figure S1. Overview of the synthesis of PAA with different cross-linker concentrations. Acrylic acid was used as a monomer. Ethylene glycol diacrylate, a bifunctional monomer, was used as a cross-linker (concentration: 0.1 mol%, 1.0 mol%, 5.0 mol%). Acrylic acid monomer and α,α'-Azobisisobutyronitrile (AIBN, KANTO CHEMICAL CO., INC.) were dissolved in a mixture of Ethyl acetate and Ethylene carbonate (mixture ratio 7:3) and bubbled with nitrogen. The mixture was bubbled with nitrogen for 30 minutes. The reaction was then carried out at 60°C for 3 hours to obtain spherical PAA particles. The resulting particles were purified by centrifugation in acetonitrile three times. After that, the solvent was removed by vacuum drying to obtain cross-linked PAA particles.
